# Selection of a Single Domain Antibody, Specific for an HLA-Bound Epitope of the Mycobacterial Ag85B Antigen

**DOI:** 10.3389/fimmu.2020.577815

**Published:** 2020-10-02

**Authors:** Paola A. Ortega, Mayra Silva-Miranda, Alfredo Torres-Larios, Eduardo Campos-Chávez, Kees C. L. C. M. Franken, Tom H. M. Ottenhoff, Juraj Ivanyi, Clara Espitia

**Affiliations:** ^1^Departamento de Inmunología, Instituto de Investigaciones Biomédicas, Universidad Nacional Autónoma de México, Ciudad de México, México; ^2^CONACyT-Instituto de Investigaciones Biomédicas, Universidad Nacional Autónoma de México, Ciudad de México, México; ^3^Department of Biochemistry and Structural Biology, Instituto de Fisiología Celular, Universidad Nacional Autónoma de México, Ciudad de México, México; ^4^Department of Infectious Diseases, University Medical Centre Leiden, Leiden, Netherlands; ^5^Center for Host-Microbiome Interactions, King’s College London, London, United Kingdom

**Keywords:** tuberculosis, single domain antibodies, peptide-human leukocyte antigen complex, mycobacterial Ag85B, T cell receptor-like antibodies

## Abstract

T cells recognizing epitopes on the surface of mycobacteria-infected macrophages can impart protection, but with associated risk for reactivation to lung pathology. We aimed to identify antibodies specific to such epitopes, which carry potentials for development toward novel therapeutic constructs. Since epitopes presented in the context of major histocompatibility complex alleles are rarely recognized by naturally produced antibodies, we used a phage display library for the identification of monoclonal human single domain antibody producing clones. The selected 2C clone displayed T cell receptor-like recognition of an HLA-A*0201 bound _199_KLVANNTRL_207_ peptide from the Ag85B antigen, which is known to be an immunodominant epitope for human T cells. The specificity of the selected domain antibody was demonstrated by solid phase immunoassay and by immunofluorescent surface staining of peptide loaded cells of the T2 cell line. The antibody affinity binding was determined by biolayer interferometry. Our results validated the used technologies as suitable for the generation of antibodies against epitopes on the surface of *Mycobacterium tuberculosis* infected cells. The potential approaches forward the development of antibody in immunotherapy of tuberculosis have been outlined in the discussion.

## Introduction

Current problems of Tuberculosis (TB) control are due to the emergence of drug resistant strains, the failure of the existing BCG vaccination and the persistence of factors associated with poverty-stricken populations. Consequently, there is a global morbidity of 10 million people with the mortality of 1.2 million in HIV-negative people and 0.25 million in HIV positive subjects (1) New approaches toward the control of TB involve the shortening of the current chemotherapy regimen (2) and prophylaxis for HIV-related TB without interfering with antiretroviral therapy (3). This pilot study, aiming at the immunotherapy of TB had an initial objective to identify monoclonal antibodies with T cell receptor (TCR)-like specificity, binding to peptide epitope/human leukocyte antigen (HLA) class I complexes on the surface of *M. tuberculosis* infected cells. This approach has been based in the evidence, that such antibodies can be developed for the specific killing of malignant and virus-infected cells (4–10).

The mycobacterial peptides epitopes complexed with MHC class I molecules on the surface of infected cells are known to be recognized and leading to the activation of CD8+ T cells (11–13). Therefore, antibodies with TCR-like specificity following conjugation with suitable apoptosis-inducing ligands could potentially become mycobactericidal and a suitable adjunct to the chemotherapy of TB. The identification of TCR-like antibodies with specificity against *M. tuberculosis* Acr1 peptides/HLA.A*0201, HLA.A*011 and HLA.A*24 class I complexes has recently been reported, using peptide/MHC complexes generated *via* UV-induced peptide exchange. The complexes were panned against human DAb (domain antibody) phage display library (14, 15). This approach can be expanded by testing immunodominant epitopes from other *M. tuberculosis* antigens recognized in the context of HLA class I alleles (16, 17). Our interest focused on the HLA-A*0201 restricted CD8+T-cell epitopes of Ag85B, a major secreted *M. tuberculosis* protein. Two Ag85B peptides _143_FIYAGSLSA_151_ and _199_KLVANNTRL_207_ had previously been identified in healthy humans and in immunized HLA-A2 transgenic mice (18), while HLA-A*0201 allele specific recognition of _37_YLLDGLRAQ_45_ and _199_KLVANNTRL_207_, was observed in patients with active TB (19). Since epitopes from Esat-6 and TB10.4, are recognized in the context of various HLA-A alleles, the HLA-A*0201 restricted, _199_KLVANNTRL_207_ epitope from Ag85B appeared to be the most immunodominant (17) and was therefore chosen as our target for the selection and characterization of a TCR-like antibody. Using a human DAb phage display library, we selected a clone producing a single domain antibody (sdAb) against the Ag85B_p199-207_/HLA-A*0201 complex (Ag85Bp/HLA-A*0201). Its specificity was determined by ELISA, and by its binding capacity to the Ag85Bp/HLA-A*0201 expressed on cells of the human HLA-A*0201 positive T2 cell line.

## Materials and Methods

### *Mycobacterium tuberculosis* Peptides/HLA-A*0201 Complexes

Three nonamer peptides, known to be HLA-A*0201 restricted CD8+T cell epitopes from: Ag85B (_199_KLVANNTRL_207_), Esat-6 (_82_AMASTEGNV_90_), or Acr1 (_120_GILTVSVAV_128_) proteins of *M. tuberculosis* (16) were synthesized by Anaspec, Inc. (USA). The peptides were of > 90% purity, and their homogeneity was confirmed by analytical reverse-phase high-performance liquid chromatography. The biotinylated recombinant complexes of Ag85Bp/HLA-A*0201, Esat-6_p82-90_/HLA-A*0201 (Esat-6p/HLA-A*0201), and Acr1_p120-128_/HLA-A*0201 (Acr1p/HLA-A*0201), were produced using extracellular HLA class I molecules, with C-terminal BirA recognition site, and β2-microglobulin (20). The insoluble aggregates expressed in *Escherichia coli* in the form of inclusion bodies were solubilized in urea and folded with peptide by dilution. Monomers were biotinylated using the BirA enzyme and purified by gel filtration on a Hiload 16/60 Superdex 75 prep grade.

### Evaluation of Refolding of Peptide/HLA-A*0201 Complexes by ELISA

An ELISA was carried out to evaluate the correct conformation of the complexes by using the W6/32 mAb (Invitrogen/USA) which recognizes a conformational epitope on the intact heavy chain/β2microglobulin complex (21–23). Briefly, 0.5 μg/well of the biotinylated complexes in Phosphate Buffer Saline (PBS), were immobilized on streptavidin coated (ThermoScientific/USA) and on uncoated high protein-binding (ThermoScientific) plates. Samples were incubated overnight (ON) at 4°C. Next day, plates were washed twice with PBS and then incubated 1 h at room temperature (RT) with W6/32 mAb diluted 1/2,000 in PBS-tween-20 0.05%, Bovine Serum Albumin BSA 2% (PBS-TBSA). After 3 washes with PBS-T, wells were incubated by 1 h with Horseradish peroxidase conjugated goat anti-mouse IgG H+L antibody (anti-mouse IgG-HRP)[1/2,000 (Invitrogen/USA)]. Then, complexes on both streptavidin coated and uncoated plates, were incubated with streptavidin-HRP (Biosource/China), diluted 1/4,000 1 h at RT. Finally the reaction was revealed with TMB (3,3’,5,5’-tetramethylbenzidine) (ThermoScientific), and stopped with 100 μl of 0.16M H_2_SO_4._ Absorbance values were measured at 450 nm using an ELISA plate reader (Multiskan Go, Thermo).

### Panning of the Human Single Domain Antibody Phage Library Against the Ag85B_p199-207_/HLA‐A*0201 Complex

A human DAb phage display library, containing approximately 3×10^9^ sdAb clones (Geneservice, Cambridge) was used following a modified protocol (24). A negative panning was carried out for elimination of the background reactivity against streptavidin as follow: 5 x10^12^ phage library particles were pre-incubated at 4°C for 1h with 30 μl of streptavidin magnetic beads M280 (Invitrogen/Norway), then tube was placed in a magnet and phage supernatant was incubated with a 7.5 μg of biotinylated Ag85Bp/HLA-A*0201 in PBS at 4°C for 1h. After that, 200 μl of streptavidin beads were added and sample was incubated for 15 min at 4°C with shaking. Beads were pulled down with the magnet and washed 15 times with PBS-T 0.1%. Finally, sdAb phage complexes were eluted by incubation with glycine–HCl pH 2.2 for 15 min at RT and sample was neutralized with Tris-HCl pH 9.0. For the second and the third round of selection, 2.5 and 1.25 μg of complexes were exposed to streptavidin beads and washed 15 and 25 times with PBS-T 0.1% respectively. After the third final round of panning, the eluted sdAb phages were used to infect 5 ml freshly prepared *E. coli* TG1 culture. Bacteria were plated onto TYE medium supplemented with 4% glucose and carbenicillin 100 μg/ml (TYG_4%_C_100_). After ON culture, 94 individual clones were picked onto a 96 wells plate, containing 200 μl of 2xTYG_4%_C_100_. Plates were incubated ON at 37°C, with shaking at 200 rpm. Next day, 5 μl of ON culture from each well was transferred to a new plate with 200 μl of fresh 2xTYG_4%_C_100_. After 3 h of culture at 37°C, 50 μl 2xTY supplemented with 4x10^8^ M13 phage was added to each well and plates were incubated for 1 h at 37°C. After centrifugation to 2,800 rpm during 10 min, pellet was re suspended in 200 μl of 2xTYC_100_K_100_ (Kanamycin 100 μg/ml). Cultures were grown at 26°C with shaking at 250 rpm, during 16-24 h.

### ELISA Phage

Phage supernatants from each well were collected and evaluated by ELISA. Biotinylated complexes at 0.5 μg/well were bound to coated streptavidin plates as described before, and incubated with 100 μl of phage supernatant for 1 h at RT. After several washes with PBS-T 0.05%, wells were incubated with anti-M13 HRP antibody (1/2,500) for 1 h at RT (GE Healthcare/USA). The reaction was developed with o-Phenylenediamine dihydrochloride OPD (SigmaAldrich/USA), and stopped adding 25 μl of 3M H_2_SO_4._ Absorbance values were measured at 492 nm using an ELISA plate reader Multiskan–GO. Phage supernatants from positive clones were also evaluated by phage ELISA against non-target complex; Esat-6p/HLA-A*0201 and Acr1p/HLA-A*0201 as described above.

### Domain Antibody Sequencing

Double strand phagemid DNA extraction was performed from 3 selected clones in *E. coli* strain TG1 by using GeneJET Plasmid Miniprep kit (ThermoScientific/Lithuania), The primers used for sequencing were LMB3 (5′ CAGGAAACAGCTATGAC 3′) and pHEN (5′CTATGCGGCCCCATTCA 3′). Sequencing was carried out in the sequencing facility at Instituto de Investigaciones Biomédicas. For translation BioEdit 7.2 software was used and BLAST and Clustalw tools for sequences analysis and alignment.

### Production of Soluble Domain Antibody

Transformation of *E. coli* HB2151 by phage infection and expression of sdAb were done with minor modifications according to (25). Once bacteria were transformed with phages, the positive clones (2C, 3C, and 7E), 50 μl of 1:10^12^ to 1:10^6^ cell dilutions were sub-cultured in TYEC_100_ plates and incubated overnight at 37°C. Three random unit forming colony (UFC) were picked up from each sample and inoculated in a culture flask containing 2xTYC_100_. The culture was grown with shaking (250 rpm) at 37°C until OD_600nm_=0.6 (26). Then, Isopropyl-β-D-1-thiogalactoside (IPTG) (Promega/USA) was added to a final concentration of 1 mM, culture was continued, at 26°C with shaking (250 rpm), ON. Cells were harvest by centrifugation at 4,500 rpm, and bacterial sediment was treated with an osmotic buffer (750 mM sucrose, 100 mM Tris pH 7.5) as described by (27). The periplasmic fractions obtained from each clone were subjected to affinity chromatography on Protein-A-agarose (Roche/Germany), in order to purify the sdAbs following the manufacturer protocol. The eluted protein fractions were shuffled and concentrated to 500 μl in PBS pH 7.4, using amicon-15ml, 10.000 WM (Merck/Ireland). Protein quantification was determined by BCA assay (Pierce/USA).

### SDS-PAGE and Western Blot

Ten μg/well of recombinant periplasmic extracts and 1.4 μg/well from 2C and 7E clones, were resolved on pre-made SDS-PAGE 4%–20%, (ThermoScientific/USA) and transferred to PVDF membranes. After 1 h blocking with PBS-BSA at RT, membranes were incubated with anti-c-Myc mAb (Sigma/USA) diluted 1/750 in PBS-T-BSA and then after washes with PBS-T, membranes were incubated with anti-mouse IgG HRP diluted (1/2,000)(Invitrogen/USA) for 1 h, washed with PBS-T, and developed with 3 mg/ml of 3,3-diaminobenzidine in PBS and 30% hydrogen peroxide diluted 1:1,000.

### Evaluation of Specificity of Single Domain Antibodies by ELISA

The specificity of the purified sdAb 2C and 7E, were evaluated by ELISA using 1μg of target Ag85Bp/HLA-A*0201 and non-targets Esat-6p/HLA-A*0201 and Acr1p/HLA-A*0201 as which were immobilized on streptavidin plates. Complexes were incubated with 5 μg of sdAb followed by incubation with 1/1,000 dilution of anti-c-Myc Ab labelled with HRP (Sigma-Aldrich/Ireland Ltd) by 1h. After several washes with PBS-T, the reaction was developed with 50 μl of TMB. The reaction was stopped with 1M of H_2_SO_4_. OD_450nm_ was measured in an Infinitum F50 microplate ELISA reader (Tecan/Switzerland). Three experiments were carried out by duplicated for 2C and 2 experiments for 7E.

### *Ex Vivo* Specificity of Single Domain 2C on the Surface of T2 Cells

To assess the ability to sdAb 2C to recognize the Ag85Bp/HLA-A*0201 an *ex vivo* assay was performed, by using the HLA-A*0201 positive T2 lymphoblastic human cell line (kindly donate by Dr. Patricia Gorocica INER-México). These cells are characterized by export empty HLA class I molecules due to a processing defect by homozygous deletion of the MHC class II region located on chromosome 6 including the TAP1 and TAP2 (transporters associated with antigen processing) genes which encode the transporter proteins (28). Cells were maintained in RPMI-1640 medium supplemented with 20% (vol/vol) fetal bovine serum (FBS) (Gibco/USA), at 37°C, 5% C0_2_. T2 cells (6x10^5^) were placed on flat bottom, 24 well cell culture plates (Costar/USA) in RPMI free serum in absence and presence of peptides from Ag85B and Esat-6.

In order to confirm the presence of HLA-ABC on T2 cells, they were incubated with W6/32 mAb (1μg/million cells), for 30 min on ice, after 3 washes, goat anti-mouse IgG Alexa fluor 488 (Invitrogen/USA) at 1/2,000 dilution was added. Samples were fixating with 0.5% PFA and the slides were mounted with vectashield (Vector Laboratories/USA). Then, cells (6x10^5^) were incubated with 80 μg of either Ag85Bp_(199-207)_ target peptide and Esat-6p_(82-90)_ as non-target. Twenty μg/ml of β2m (Sigma) was added according to (29). Cells were incubated for 8 h at 37°C in 5% CO_2_ atmosphere, after that cells were washed twice with PBS and incubated with 10 μg of sdAb 2C ON at 4°C in agitation, and then washed 2 times, with PBS, following by incubation with anti-c-Myc Ab (Santa Cruz-/Europe) (1/100) for 1 h at RT. After three washes, anti-mouse IgG Alexa fluor 488 (Invitrogen/USA) (1/2,000) was added and samples were incubated for 1 h at RT. After three washes, Hoechst 33343 (Life technologies/USA) diluted 1/9,000 was used for 10 min for nucleus staining. sdAb 2C was evaluated on cells in the absence of peptides and as a staining control in one condition, no domain was added. Fluorescence images were acquired with Olympus BX41, (Fluorescence-Microcopy) using the 100x magnifying lens, the digital images were captured with Zen 2.6 blue edition software, and the capture parameters, exposure time and intensity for each staining system, were applied in both control and problem samples.

### Kinetic Binding Assays for the Assessment of the Interaction of sdAb 2C With Ag85B/HLA-A*0201 Complex by Biolayer Interferometry

The binding kinetics and the determination of the dissociation constant (*K_D_*) for the sdAb 2C against the Ag85Bp/HLA-A*0201 complex were performed using Biolayer Interferometry (BLI) at 25°C. Streptavidin biosensors in an Octet RED96 system (FortéBio Inc. San Jose, CA, USA) were used. The assays were performed on black bottom 96-well microplates (Greiner Bio-One 655209) in a total volume of 200 μl with orbital shaking at 1000 rpm. Experiments were controlled with the software Data Acquisition 8.2 (ForteBio, Inc.) For the BLI experiment, a baseline was established using 1x Kinetics buffer (FortéBio Inc. San Jose, CA, USA). Then, the biotinylated Ag85Bp/HLA-A*0201 complex at 25 ng was allowed to bind to streptavidin sensor for 5 min, followed by washing with the same buffer to eliminate nonspecific binding. Next, the purified sdAb was bound to the Ag85Bp/HLA-A*0201 complex in the biosensor and the association rate was measured (*k_a_*). In the last step, the dissociation rate (*k_d_*) of the antibody-complex was obtained. The BLI experiment was done with six different concentrations of the sdAb 2C from (2.54 to 81.3 μM), one well with 200 μl without sdAb was used as a negative control. New streptavidin biosensors were used for each experiment. The binding of sdAb 2C at 10 μM, to non-target Esat-6p/HLA-A*0201 was also tested. The data were processed using the Octet Data Analysis Software version 8.2 (FortéBio Inc. San Jose, CA, USA) according to a 1:1 model.

### Statistical Analysis

GraphPad Prism version 6.0c software was used to analyze the results. For the statistical analysis, one-way ANOVA multiple comparisons with Sidak`s post Hoc correction was used.

## Results

### Evaluation of Peptide HLA-A Complexes With W6/32 Antibody

**Figure 1:**
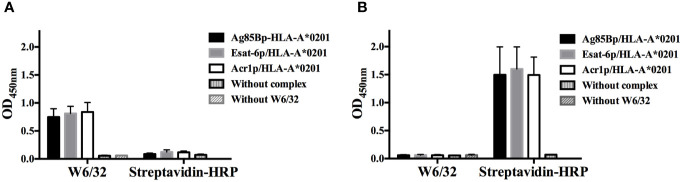
Evaluation of complexes with conformational antibody W6/32. **(A)** Two groups of biotinylated complexes were immobilized on streptavidin coated plates, the group on the left was detected with W6/32 and the group on the right, was incubated with HRP-streptavidin. **(B)** The same as A, but both groups of biotinylated complexes were bound to uncoated streptavidin plates and detected with W6/32 and HRP-streptavidin respectively. The dates represent the media +/- standard deviation from three independent experiments.

Ag85Bp/HLA-A*0201, Esat-6p/HLA-A*0201, and Acr1p/HLA-A*0201 immobilized on streptavidin plates were recognized by W6/32 mAb (**Figure 1A**), indicating this result that peptides HLA-A*0201 complexes were correctly folded. In contrast, the mAb did not recognize the complexes bound to non-coated streptavidin control plate (**Figure 1B**), showing the results that direct binding of the complexes in the plates could lead to a loss of conformation. The biotinylated complexes bound to streptavidin plates were not recognized for streptavidin-HRP, an indication that biotinylated complexes were correctly oriented by streptavidin on coated plates (**Figure 1A**). In contrast, the positive signal obtained with the complexes bound to the non-coated streptavidin plate (**Figure 1B**), was an indication that exposed biotin in unfolded complexes was being recognized by streptavidin-HRP.

### Screening of the Human Single DAb Phage Library Binding to Ag85B_p199-207_ HLA‐A*0201/Complexes

**Figure 2:**
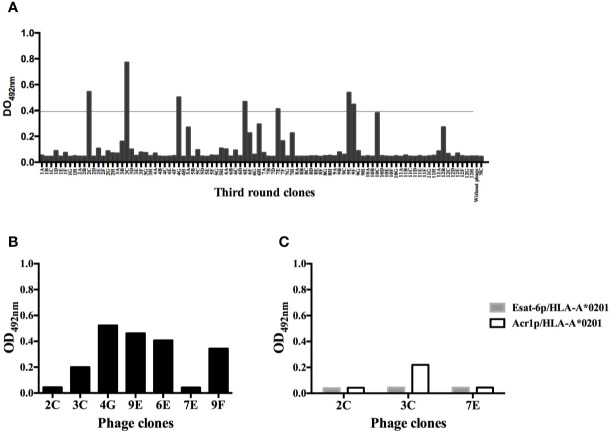
Monoclonal phage-domain Immunoassay. **(A)** Clones from the third round of panning. Positives clones showed signal recognition to Ag85Bp/HLA-A*0201 of 10 fold higher than negative control (NC), well with unrelated sdAb. Line indicates the threshold used to define a positive result. **(B)** Positive clones exposed to streptavidin coated plates (without complex). **(C)** Clones 2C, 3C, and 7E that showed the lowest recognition signal towards streptavidin tested with non-target peptides.

The number of phage particles from sdAb library during the three rounds of selection was consistent with the published protocol (24). The final output titers of phage particles showed an enrichment factor of 25 (**Table 1**). From 94 clones evaluated by monoclonal phage-ELISA, only 7 clones showed absorbance 10 fold higher than the negative control (clone not reactive to the Ag85Bp/HLA-A*0201) (**Figure 2A**). From those, clones 2C, 3C, and 7E did not recognized streptavidin (**Figure 2B**) and all of them showed specific binding by ELISA to the target complex and but none bound to non-target complexes, Esat-6p/HLA-A*0201, and Acr1p/HLA-A*0201 (**Figure 2C**).

**Table 1:** Selective enrichment of phage domain antibody after 3 rounds of biopanning.

SelS SelectionRound	Ag85Bp/HLA-A*0201	Phages Input	Eluted phages/ml	Eluted/input	EnrichmentFactor*	AmplifiedPhages/ml	Tween 20/ # washes
1	7.5μg	5.0×10^12^	6.0×10^6^	1.2×10^-6^	1.0	84×10^12^	1.0%/15
2	2.5μg	5.0×10^12^	1.4×10^8^	2.8×10^-5^	23	7×10^12^	0.1%/15
3	1.25μg	5.0×10^12^	1.5 ×10^8^	3.0×10^-5^	25	—	0.1%/25

*Enrichment factor was determined by dividing the eluted/input ratio in each round by the ratio in the first round. according to Bagheri et al. (30) Zhang et al. (31).

### Domain Antibody Sequencing

BLAST search analysis of sdAb 2C, 3C, and 7E sequences, showed that all them matched with 122 amino acids length of immunoglobulin heavy chain variable region, partial (>ABM67233.1 Homo sapiens). Sequences corresponded to a human dAb with a length of 160 and 159 amino acids residues for 2C and 7E domains, respectively, the protein sequence of the 3C domain was exactly the same as 2C but shorter in length, 3C had only 151 amino acids due to the presence of a stop codon. For all sequences, the three complementarity determining regions (CDR) and the c-Myc tag sequence were identified. Clones 2C and 7E were selected to continue with de antibody expression phase.

### Production of Single Domain Antibodies

**Figure 3:**
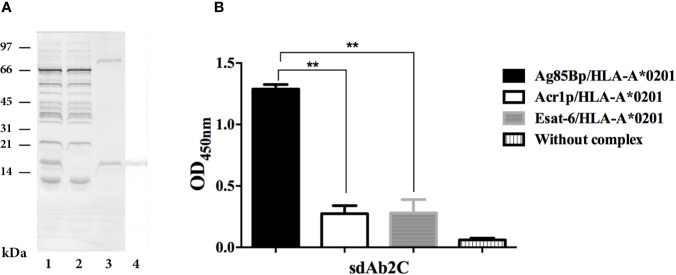
Expression, purification and specificity evaluation of sdAb 2C. **(A)** Lanes 1 to 3. Line 1, Coomassie blue stained of periplasmatic extract. Line 2, unbound fraction to protein A sepharose column. Line 3, purified domain. Lane 4, Western blot of purified sdAb 2C recognized by anti-c-Myc Ab. **(B)** Evaluation of specificity of sdAb 2C by ELISA with target Ag85Bp/HLA-A*0201 and non-target (Esat-6p/HLA-A*0201 and Acr1p/HLA-A*0201) complexes. The results represent 3 independent experiments and significant differences are indicated by asterisk (p < 0.05).

sdAb 2C was produced in *E. coli* HB2151. The expression and purification of sdAb 2C is shown in **Figure 3A**. Purified sdAb 2C with the expected molecular mass of ≈15 kDa was detected by Coomassie blue staining (**Figure 3A**, line 3) and antibody was recognized by anti-c-Myc Ab on Western blot (**Figure 3A**, line 4). The yield of production of 2C was 1418 µg from 50 ml of culture, (Results obtained with 7E are not shown).

### Specificity of Single Domain Antibodies by ELISA

The recognition of the Ag85Bp/HLA-A*0201 complex by sdAb 2C was evaluated by ELISA, using Esat-6p/HLA-A*0201 and Acr1p/HLA-A*0201 as non-targets. The results are shown in **Figure 3B**. The binding of sdAb 2C to Ag85Bp/HLA-A*0201 complex was highly specific, showing statistically significant differences with respect to non-targed complexes. On the other hand, sdAb 7E, showed a nonspecific signal absorbance ratios for both target and non-target complexes (Results not shown).

### *Ex Vivo* Specificity of Soluble Domain 2C on T2 Cells Surface

**Figure 4:**
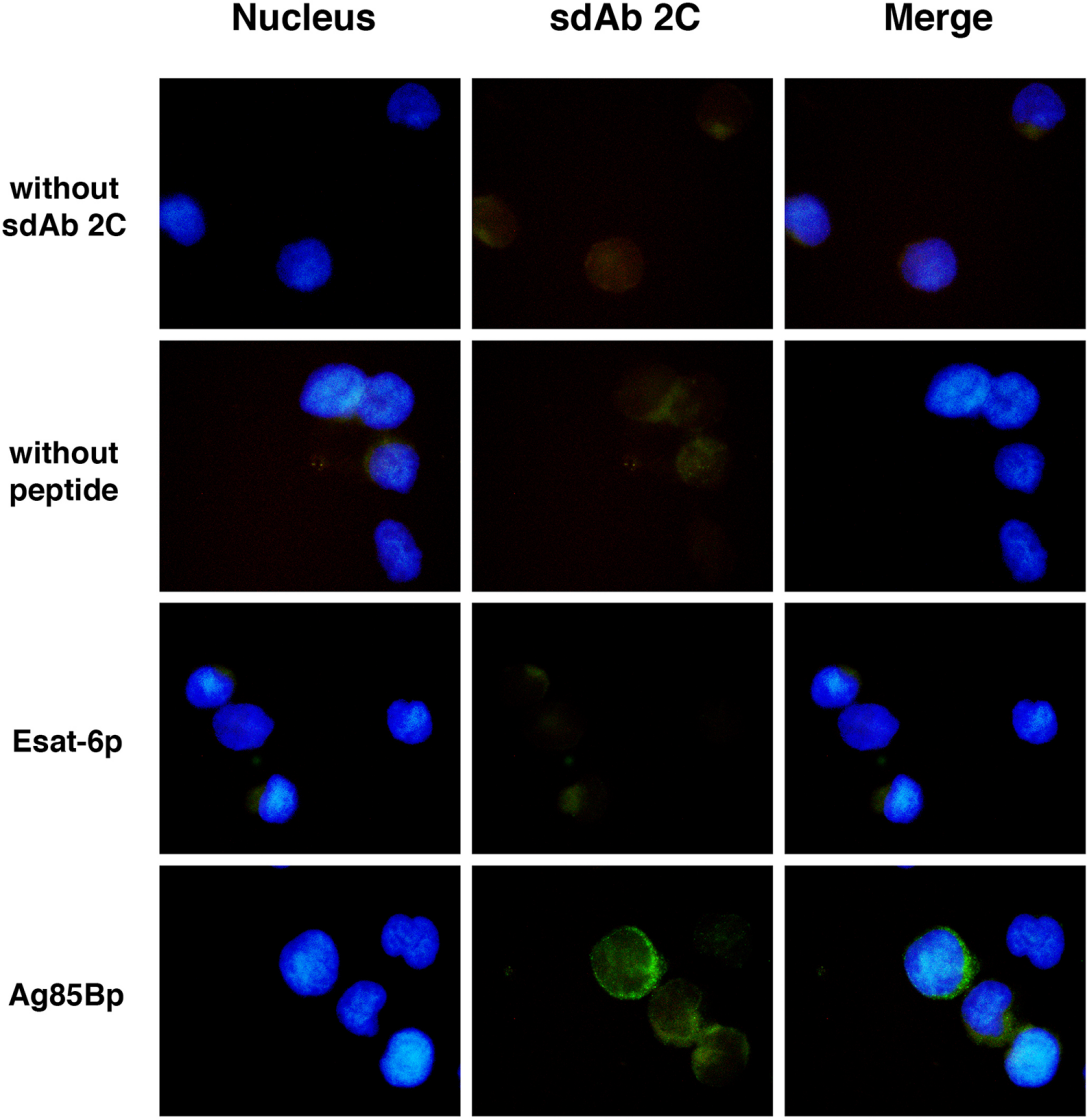
Recognition of *M. tuberculosis* peptide/HLA-A*0201 complex on T2 cells surface by sdAb 2C. Cells were exposed to Ag85B and Esat-6 peptides. As negative control cells without peptide were used. Peptide/HLA-A*0201complexes were detected with sdAb 2C follow by anti-IgG coupled to Alexa Fluor 488 as secondary Ab. More than 200 fields were examined for each condition using the 100x magnification, by fluorescence microscopy.

Surface expression of HLA-A molecules in T2 cells was demonstrable by binding of the W6/32 mAb in absence and in presence of either Ag85Bp_199-207_ or Esat-66_p82-90_ peptides (**Supplementary Figure S1**). However, sdAbs 2C showed different recognition patterns, whereby the sdAb 2C fluorescence signal was observed only on T2 cells exposed to the Ag85Bp_199-207_ (**Figure 4**). About 6.4% of positive events were observed, but no positive signals were detected without sdAb 2C, or without Ag85Bp_199-207_, or by incubation with the no-target Esat-6 peptide.

### sdAb 2C Binding Affinity by Biolayer Interferometry

**Figure 5:**
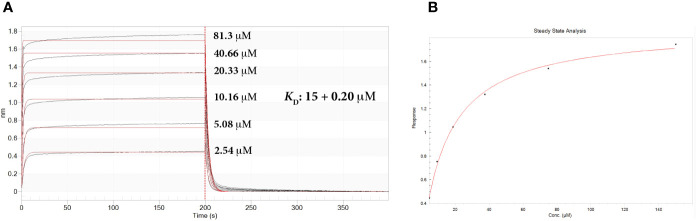
Real-time biolayer interferometry sensorgrams for determination of the binding affinity. **(A)** Sensorgram showing reference substrate binding, on streptavidine biosensors, of Ag85Bp/HLA-A*0201 complex with increased concentrations of sdAb 2C. Calculated affinity constant *K*_D_ is shown in the upper right of the sensorgram. Association constant, *k_a_*: 1.93 × 10^4^ + 0.02 × 10^4^ M^-1^ ^s-1^; dissociation constant, *k_d_*: 0.289 + 0.003 s^-1^. **(B)** Steady state analysis of the binding response (nm) as a function of sdAb. Calculated *K*_D_ 15.0 + 0.7 μM.

The association of sdAb 2C to Ag85Bp/HLA-A*0201 was measured by BLI. The affinity constant was calculated in terms of equilibrium dissociation constant (*K*_D_) to be 15 + 0.20 μM (**Figure 5A**). The sdAb interacts with the target complex in a concentration dependent manner and confirms the dissociation constant value (**Figure 5B**). The interaction of sdAb with Esat-6p/HLA-A*0201 was very low and the binding parameters could not be determined.

## Discussion

CD8+ T cells recognizing peptide epitopes bound to MHC/HLA class I molecules have the capacity to lyse the *M. tuberculosis* infected cells, thus contributing to the intracellular killing of the infecting organisms (16). Hence, binding of antibodies with TCR-like recognition specificity seemed desirable for developing antibody constructs, with mycobactericidal potentials. TCR-like Ab recognizing MHC class I bound antigenic peptides on antigen presenting cells, have previously been reported for the treatment of cancer, viral infections and autoimmune diseases (6). The aim toward TB immunotherapy has recently been initiated by the selection of antibodies against the latency expressed Acr1/HLA class I restricted epitopes (14, 15). We report here on a TCR-like sdAb against an immunodominant HLA-A*0201 binding epitope of the Ag85B, selected by screening and selection from a human DAb phage display library. The secreted, fibronectin binding, mycolyl transferase protein is a strong immunogen in both infected and active TB cases and it has been used in several recombinant vaccine constructs (32–35). We chose the _p199_KLVANNTRL_207_ peptide as the target epitope in this study, because its known immunodominance for the human CD8+ T cell responses in the context of HLA-A*0201 and it’s a conserved sequence in the genome (16, 17, 19). These properties favoured the previous application of TCR-like sdAbs for targeting tumors and cells infected with other pathogens (36, 37).

The procedures used for the selection and evaluation of TCR-like sdAbs ensure the specificity of recognition between for the target and non-target molecules. In this work, all the biotinylated complexes were first evaluated through a comparative ELISA, using plates with or without streptavidin. The results showed that the streptavidin coated plates ensured an adequate arrangement and orientation of p/HLA-A complexes, which is necessary for finding the specific TCR-like sdAbs. Similarly, Ag85Bp/HLA-A*0201 was bound to magnetic pearl cover with streptavidin for the selection of recombinant phages (26). From the third-round of selection, clones, 2C and 3C of identical sequence and clone 7E bound to the target complex. However, after conversion to the soluble form, sdAb 7E lost its specificity for the target complex. Such a change in specificity was previously reported to be due to loss of structural support by the phage scaffold pIII protein for the anti-H1N1 influenza virus antibody’s antigen-binding site (38). The production of sdAb 2C and 7E in *E. coli* HB2151 from periplasmatic extracts was satisfactory, compared with the previously reported production outputs (25, 39).

Although sdAb 2C was highly specific against the target complex, its affinity is low, i.e., in the range of 1–100 μM corresponding to the affinity binding interaction of TCR ligands. The low binding affinity known for TCR-like antibody fragments selected from human libraries can be improved using complementary technologies (40). An increased up to 100 fold of the initial affinity from scFv (single chain variable fragment) directed against the HLA-A2-pWT1_126_ complex, was achived by mutagenesis combined with yeast display based on one specific scFv-clone (41). The affinity could also be improved by re-cloning and re-selection of clones or by conversion of sdAbs into multivalent formats with higher avidity (4, 42). Though antibody affinity can be significant for its potential for immunotherapy, most interestingly however, it was found that low, rather than high antibody affinity has been reported to essential for the passive antibody therapy of a drosophila based model of Alzheimer’s disease (43, 44). This was interpreted on the grounds that the low affinity anti-tau antibody may loosen up intracellular tau aggregates allowing better access of lysosomal degrading enzymes, while high affinity antibody may make these aggregates more compact and therefore more difficult to degrade. Such interpretation may be relevant also for the desired intracellular mycobactericidal action on *M. tuberculosis* infected macrophages.

Antibodies generated in animals against MHC-I recombinant tetramers are rarely TCR-like, because many of them recognize the α3 domain of MHC-I and β2 microglobulin (β2m) (5) and also due to the reduced stability of recombinant p/HLA-A complex (45). However, the phage display antibody libraries have the advantage that the fusion proteins are exposed on the surface of phage particles, and recombinant p/HLA-A complex targets can be used through several selection rounds (37). The structure of human sdAbs also known as nanobodies, used in the present work represents a single variable domain based on the VH3-23 germline segment heavy chain with synthetic diversity introduced by PCR mutagenesis into all tree complementary determining regions (24). They are highly stable, easily produced in large quantity by *E. coli*, they are of low immunogenicity, small size (15 kDa), and can be fused with multiple tags (46–48). Consequently, sdAbs have been used for virus detection (49–51), for imaging *in vivo*, mainly in cancer due to their bio distribution, high tumor penetrance and fast clearance from the blood circulation (52). The immunotherapeutic potentials include therapeutic targets in cancer (53), antagonism of angiogenesis (54) and acting as metastasis inhibitors.

## Conclusions and Perspectives

The TCR-like identity of the sdAb 2C has been validated by its specific recognition of Ag85Bp/HLA-A*0201 on the surface of human T2 cell line. It will be of further interest to test, if the sdAb 2C can detect the expression of the HLA-A*0201-bound Ag85B peptide on human macrophages, which contain either replicating or dormant *M. tuberculosis *infection. A positive result would justify further engineering of the sdAb 2C to become an immunotoxin, by conjugation with suitable apoptosis inducing ligands,* *i.e., *Pseudomonas* exotoxin A, Granzyme B or BH3 peptide (55, 56), in endeavour to develop a mycobactericidal immunotherapeutic agent.

Further, development of sdAb 2C will need evaluation in both HLA-A*0201 transgenic mice and in humans with active TB disease. The obtained results are showing the feasibility of selecting TCR-like sdAbs to other immunodominant epitopes of *M. tuberculosis* and their future development as potential immunotherapeutic adjuncts to the chemotherapy of TB.

## Data Availability Statement

The raw data supporting the conclusions of this article will be made available by the authors, without undue reservation.

## Author Contributions

CE and JI conceived and supervised the study. CE, PO, MS-M, and KF performed experiments. TO, KF, MS-M, and PO designed experiments, analyzed data, and provided new tools and reagents. CE, JI, and PO wrote the manuscript. All authors contributed to the article and approved the submitted versión.

## Conflict of Interest

The authors declare that the research was conducted in the absence of any commercial or financial relationships that could be construed as a potential conflict of interest.

## Supplementary Material

Supplementary Figure 1 Recognition of HLA-A molecules on T2 cell surface. Cells were loaded with Ag85B and Esat-6 peptides and cells without peptide as negative control. HLA-A molecules were detected with W6/32 as primary Ab, by anti c-Myc Ab and anti IgG coupled to Alexa Fluor 488 as secondary Abs. More than 200 fields were observed for each condition evaluated using the 100x magnification, by fluorescence microscopy.
