# Supplementary figures and images for: Selection of a Single Domain Antibody, Specific for an HLA-Bound Epitope of the Mycobacterial Ag85B Antigen
Source: Front Immunol. 2020 Oct 2;11:577815. doi: 10.3389/fimmu.2020.577815 (PMC7564862; doi:10.3389/fimmu.2020.577815)

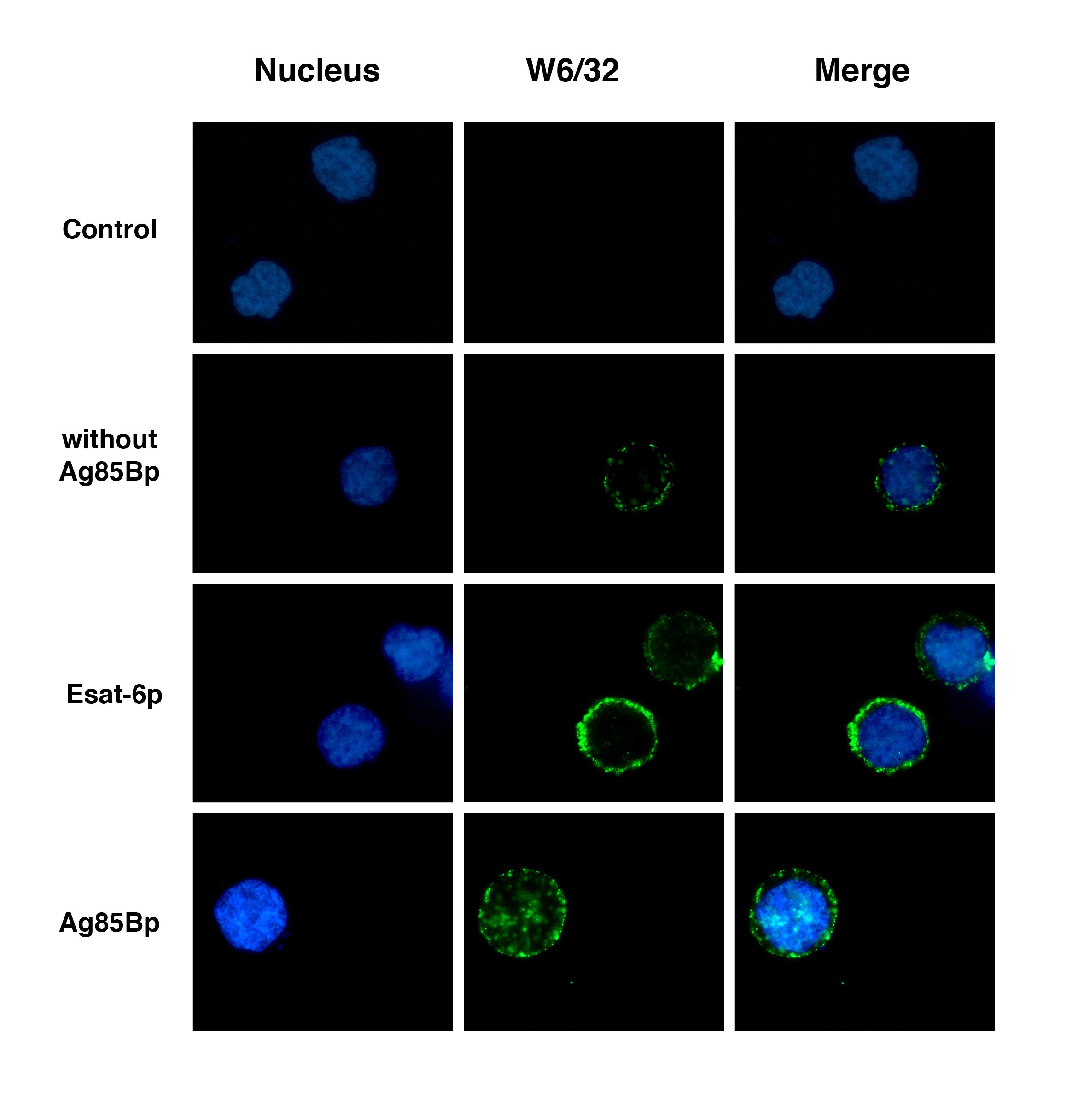

Supplement: Supplementary Figure 1 — Recognition of HLA-A molecules on T2 cell surface. Cells were loaded with Ag85B and Esat-6 peptides and cells without peptide as negative control. HLA-A molecules were detected with W6/32 as primary Ab, by anti c-Myc Ab and anti IgG coupled to Alexa Fluor 488 as secondary Abs. More than 200 fields were observed for each condition evaluated using the 100x magnification, by fluorescence microscopy. [file Image_1.jpeg]
